# Food By-Products and Agro-Industrial Wastes as a Source of β-Glucans for the Formulation of Novel Nutraceuticals

**DOI:** 10.3390/ph16030460

**Published:** 2023-03-20

**Authors:** Diego Morales

**Affiliations:** 1 Nutrigenomics Research Group, Department of Biochemistry and Biotechnology, Universitat Rovira i Virgili, 43007 Tarragona, Spain; diego.morales@ucm.es or diegomoraleshdz@gmail.com; 2 Departmental Section of Galenic Pharmacy and Food Technology, Veterinary Faculty, Complutense University of Madrid, 28040 Madrid, Spain

**Keywords:** food by-products, agro-industrial wastes, β-glucans, antioxidant, hypocholesterolemic, cereals, mushrooms, nutraceuticals

## Abstract

Food and agro-industrial by-products provoke a great environmental and economic impact that must be minimized by adding value to these wastes within the framework of circular economy. The relevance of β-glucans obtained from natural sources (cereals, mushrooms, yeasts, algae, etc.), in terms of their interesting biological activities (hypocholesterolemic, hypoglycemic, immune-modulatory, antioxidant, etc.), has been validated by many scientific publications. Since most of these by-products contain high levels of these polysaccharides or can serve as a substrate of β-glucan-producing species, this work reviewed the scientific literature, searching for studies that utilized food and agro-industrial wastes to obtain β-glucan fractions, attending to the applied procedures for extraction and/or purification, the characterization of the glucans and the tested biological activities. Although the results related to β-glucan production or extraction using wastes are promising, it can be concluded that further research on the glucans’ characterization, and particularly on the biological activities in vitro and in vivo (apart from antioxidant capacity), is required to reach the final goal of formulating novel nutraceuticals based on these molecules and these raw materials.

## 1. Introduction

The growing concern in modern societies about the impact of diet composition on human health has encouraged scientists to thoroughly investigate the target compounds that can be found in food sources and their specific effects. Among these molecules, dietary fiber has attracted great attention because of its beneficial effects on human physiology and metabolism, demonstrating the ability to reduce the risk of disorders such as obesity, diabetes, intestinal diseases, cancer, etc. [1]. The term ‘dietary fiber’ is related to the edible part of plants and mushrooms that cannot be digested or absorbed in the human small intestine, being completely or partially fermented in the large intestine. It includes polysaccharides, oligosaccharides and associated substances (cellulose, hemicellulose, lignin, pectin, gums, mucilages, etc.) [1,2]. 

β-glucans are classified as viscous, soluble and fermentable fibers and are mainly present in cereals, mushrooms, algae, yeasts and bacteria [2]. They are constituted by a polymeric chain of glucose monomers that are linked by glycosidic bonds in β-(1→3) in algae and bacteria, showing a linear conformation (although some bacterial β-glucans can also show ramifications). However, branched structures can also be observed in cereals, with linkages β-(1→3) and β-(1→4) as well as in mushrooms and yeasts, with linkages β-(1→3) and β-(1→6) [2,3]. The importance of these macromolecules is related to the interesting activities that they can exert, highlighting the capability of modulating the immune system and exerting hypoglycemic and hypolipidemic effects, particularly reducing serum cholesterol levels [4,5,6]. Moreover, technological functionalities can be also exploited, since antioxidant and antimicrobial activities of β-glucans might be used in food preservation and their gelling properties have been applied, for instance, to develop easy-to-swallow products for elderly people [7,8,9]. 

Considering the different sources that contain significant amounts of β-glucans, agri-food by-products emerge as interesting raw materials that can be subjected to extraction and isolation procedures to recover these carbohydrates. Additionally, they can also be utilized as substrate for organisms that synthesize β-glucans [10,11]. In this respect, the amount of waste that is generated in agricultural processing and food industries is still growing together with world population, as well as their impact on the environment and economy [12]. Therefore, their revalorization is a key goal that must be addressed encouraging further research on technologies that allow the obtention of high-value bioactives such as β-glucans.

In the present work, the scientific literature was reviewed to identify the recent insights into the extraction, isolation and production of β-glucans from agri-food sources, focusing on by-products and wastes that are normally discarded. Studies targeting the use of these recovered molecules in the design of novel nutraceuticals and functional foods were also investigated. 

## 2. Obtention of β-Glucans from Food Sources

There is a great diversity of natural sources that have been studied because of their content or their ability to produce bioactive β-glucans (Table 1).

Among them, cereals have been considered as relevant materials to extract these polysaccharides, particularly from oat (*Avena sativa*) that normally showed contents ranging from 2–7% and barley that might even reach levels up to 11% [33,34,35]. The differences are not only influenced by species and varieties, but also by the environment: geographical distribution, soil, climate conditions, etc. Normally, wild varieties contain higher concentrations than domestic ones and, in the case of barleys, waxy and hull-less varieties seemed to be richer in β-glucans [34,36]. Both oat and barley β-glucans demonstrated the ability to reduce blood LDL cholesterol (3 g β-glucan/day) and decrease postprandial glycemic responses (4 g β-glucan/day) [37]. Other cereals such as wheat, corn or rye are also interesting sources of these polysaccharides, but their content is usually lower. In all cases, their presence in cereals and grains is related to a specific structure formed by β-linked-(1→3) and -(1→4)-glucose monomers, although characteristics such as molar mass, molar ratio, solubility or viscosity can be significantly different [34]. 

Apart from the Plantae kingdom, mushrooms and yeasts have been exhaustively investigated to isolate fungal glucans. The most representative structure is the branched (1→3),(1→6)-β-glucan, that can be found in white button, shiitake, oyster and porcini mushrooms, among others, as well as in black, summer and desert truffles [3,19,20,21,23,38]. As a matter of fact, specific structures with (1→3),(1→6)-β linkages such as lentinan and pleuran were described for *L. edodes* and *Pleurotus* spp., respectively, with potent and promising properties, e.g., immune-modulatory, antioxidant, antitumoral, antiviral, etc. [39,40]. Moreover, linear glucans were also found: (1→3)-β-glucans were observed in *P. ostreatus* and *B. edulis*, while (1→3)-β-glucans were isolated from *A. bisporus* and *L. edodes* [3,18]. These linear and branched structures are also present in yeast cell walls or produced by species related to food and food processes, with *Saccharomyces cerevisiae* being the most deeply studied yeast in fermented food and beverages, followed by other brewing yeasts such as *Saccharomyces pastorianus* but also other non-*Saccharomyces* species such as *Kluyveromyces marxianus*, mainly linked to fermented dairy products, or *Mestchnikowia pulcherrima*, with growing relevance in vinification [28,29,30,41].

Finally, the most common pattern found in bacterial β-glucans is formed by linear chains with (1→3) bonds, being observed in lactic acid bacteria (LAB) such as *Levilactobacillus brevis* [31]. However, several studies found that other LAB such as, for instance, *Oenococcus oeni*, *Lactobacillus diolivorans* and *Pediococcus parvulus* naturally produced 2-substituted (1→3)-β-glucans forming branched structures [32,42].

Attending to the wide range of matrices from which β-glucans can be recovered, different extraction and purification procedures have been tested and optimized during recent decades to obtain high yields and selectivity (Table 2).

Since these procedures are usually linked to the formulation of functional food or nutraceuticals, food grade solvents and environmentally friendly techniques are often required, with water extraction being the simplest way to obtain glucan-rich fractions. However, aqueous methods at room temperature show the ability to recover only polysaccharides with high water solubility [43,45]. Therefore, to increase extraction yield and collect insoluble glucans too, other alternatives using hot water (up to its boiling point), ethanol or alkaline solutions can be applied [44,46,64,65]. Nevertheless, the matrices that are frequently utilized as raw materials need to be subjected to more aggressive conditions such as with the aid of specific enzymes (e.g., α-amylase, xylanase, proteases, cellulase, etc.) [50,51], microwaves or ultrasounds to degrade vegetal, fungal, bacterial or algal cell walls [52,57,58] and also pressurized liquid methods to allow operations at subcritical conditions reaching temperatures above the boiling point of the extraction fluid (usually water) and leading to efficient and high-yield methods [53,59]. Moreover, other advanced and innovative technologies are being tested such as electric field-assisted extraction, that applies high-voltage pulses to increase the porosity of cell walls and facilitate bioactive recovery, although, to date, just a few works have used this technique to extract β-glucans and other polysaccharidic structures [62,63].

Depending on the selectivity grade of the extraction method, different purification or isolation processes can be added as a subsequent step to obtain high-purity β-glucans. For instance, freeze–thawing is a simple but efficient process that separates polysaccharides with different branch degrees in a cold water soluble fraction and a non-soluble pellet. Moreover, treatment with solvents (cold ethanol, dimethyl sulfoxide, sodium hydroxide or Fehling solutions, etc.) are useful to precipitate or solubilize specific glucans. Together with these widely used methods, alternatives such as closed dialysis, ultrafiltration or column fractionation based on molecular sizes have been also employed [66].

## 3. Formulations of Dietary β-Glucans with Biological Activities

The promising results of β-glucans targeting specific health conditions have placed them as great candidates for the formulation of nutraceuticals and functional food products. As was previously commented, EFSA confirmed the significant hypocholesterolemic effect of β-glucans from oat and barley. This action is mainly carried out through a reduction in cholesterol absorption by increasing viscosity in the gastrointestinal tract, stimulating lipid excretion [67]. Moreover, some authors observed that barley β-glucans could also reduce 3-hydroxy-3-methylglutaryl-coenzyme A reductase (HMGCR) activity, decreasing cholesterol biosynthesis rate, together with an upregulation of cholesterol 7-α-hydroxylase, promoting bile acid synthesis and reducing cholesterol levels [68]. Some examples of hypocholesterolemic products prepared with dietary β-glucans can be found in the market as functional foods (fortified cereals, bread, flours, cookies, etc.) or nutraceuticals, including not only glucans from cereals but also those from other sources such as mushrooms, although the health claim approved by EFSA is specifically for oat and barley [37]. A positive response was also obtained for the glucose control ability of these glucans since they are capable of reducing postprandial glycemic response. It was observed that their consumption led to a decrease in available glucose together with glucose transport inhibition and intestinal disaccharidase activities. Although the mechanism of action is not fully understood yet, the rheological properties of these polysaccharides seem to be crucial [37,69]. The main challenge of these kind of products is that the claimed effect is warranted by the consumption of 4 g of β-glucans per 30 g of available carbohydrates, so their formulation must ensure a high concentration of glucans (and low concentration of other carbohydrates) using formats such as pills or capsules or fortifying foodstuffs such as flour, flakes, pasta, bread, etc. [70].

Despite the fact that EFSA has not approved a health claim for the immune-modulatory activity of β-glucans yet, the great number of works that correlated β-glucan consumption (particularly fungal glucans because of their structure) and immune responses has motivated the formulation of a lot of nutraceuticals that can be found in the market (Glucan 300^®^, Yestimun^®^, Imunoglukan^®^, etc.) [71]. Different mechanisms are involved in this activity and are not totally understood, but it seems that these glucans can be recognized as pathogen-associated molecular patterns (PAMPs), modulating immune cells [72]. 

## 4. Use of Food By-Products and Agro-Industrial Wastes to Obtain Bioactive β-Glucans

Current difficulties related to the increase in global population, together with food availability problems or environmental threats such as climate change, are encouraging the optimization of food system structures to achieve sustainable development goals within the framework of circular economy. In this line, the utilization of food by-products and agro-industrial wastes, that are massively and constantly generated, is a key point to reduce their negative impact from not only an environmental but also an economic point of view [73,74]. 

Considering these leftovers or underutilized materials as potential sources to extract β-glucans or β-glucan-rich fractions, two main approaches should be described: in the first case, these wastes can present a significant content of these polysaccharides and therefore be subjected to extraction, enrichment, purification or isolation processes to obtain glucans; the second one is the use of the by-products as a substrate that is fermented or processed by β-glucan-producing species (Table 3).

### 4.1. Yeast Residues from Wine and Beer Industries

For instance, spent yeasts for wine and beer processing are interesting by-products that can be revalorized when subjected to the extraction of the β-glucans that are present in yeast cell walls. Related to this, wine lees contain sedimented or spent yeasts that are generated during winemaking steps (fermentation, filtration, centrifugation, aging), being the main volume produced before and after the completion of alcoholic and malolactic fermentation [76]. Varelas et al. (2016) described a process that involved autolysis of the yeasts, hot alkaline extraction and spray- or freeze-drying to obtain β-glucans from red and white wine lees [76]. A similar procedure was carried out later by Rozmierska et al. (2019) applied to apple wine lees, obtaining β-glucan-rich cell wall preparations with interesting fat- and water-binding capacities [75]. Moreover, spent yeasts from beer elaboration constitute also an important material that must be revalorized since yeast residues represent up to 15% of total by-products from brewing procedures, reaching values up to 30 g per liter of beer [77]. The high content of β-glucans in brewery yeasts (both *Saccharomyces* and non-*Saccharomyces* spp.) has motivated the application of different extraction and purification procedures including hot water and alkaline treatments, as well as dialysis, centrifugation and freeze-drying processes [10]. The obtained and tested glucans demonstrated technological functionalities as fat replacers, with promising results in food products such as yoghurts and mayonnaise [77,94], and also biological activities, acting as antioxidant and cytoprotective agents [27,95]. 

### 4.2. By-Products from Cereals and Other Vegetal Sources

Although a great volume of by-products is generated as agri-food waste from the cereal industry (bran, germ meal, middlings, husks, etc.), this material is still underexploited in terms of β-glucan recovery. However, several works revalorized, among others, barley by-products such as those discarded during mechanical processing of grains [78] or the bran obtained from hull-less barley [61]. Moreover, the resultant leftovers from oat milling were also subjected to extraction and purification procedures [79]. Among the methods applied to cereal wastes, hot water and pressurized liquids have been used to extract glucans, as well as ultrasound- and microwave-assisted extraction or ultrafiltration techniques to enrich, purify or isolate β-glucan fractions [61,79].

Apart from cereals, other plant species and their by-products have been studied to this end. For instance, pomegranate (*Punica granatum*) peel β-glucans were extracted with acetone or methanol and showed in vitro antioxidant activity against human breast and uterine cancer cell lines. However, the structure of the obtained glucans was not elucidated, and the origin of the glucans (plant, fungal, bacterial) was not described [81]. Furthermore, broad bean (*Vicia faba*) pods discarded from seeds, that were collected at different maturation stages, were subjected to a sequential procedure that included hot alkaline extraction, deproteinization and glucan precipitation with ethanol. The β-glucan extraction yield was higher at the last maturation stages [82]. Other seeds such as walnuts (*Juglans regia*) also generated an important waste because of the non-edible husks. La Torre et al. (2021) demonstrated that green husks of walnuts could be a relevant source of β-glucans [80]. 

### 4.3. Mushroom By-Products

The mushroom industry produces wastes with a significant impact on the environment and economy. Mainly, the most frequently generated ones are stipes or stalks, non-marketed caps, spent mushroom substrates and those mushrooms that are normally discarded because they do not comply with the commercial standards (size, shape, etc.) [96]. These by-products contained high levels of β-glucans, similar to those registered for edible marketed fractions [43], but just a few works have targeted these leftovers to extract glucans. In this line, Aguilo-Aguayo et al. (2017) applied ultrasound-assisted extraction methods to *Agaricus bisporus* caps and stalks, obtaining β-glucan-rich fractions [57]. Furthermore, wastes from other mushrooms such as *Pleurotus pulmonarius* stalks were treated with hot water to obtain extracts with high levels of β-glucans that exerted potent in vitro antioxidant activity, that was subsequently validated in vivo in Nile tilapia (*Oreochromis niloticus*) as a fish model [83]. These results were also confirmed for a purified polysaccharidic fraction in the same organism [84].

### 4.4. Residues Utilized as Substrates for the Growth of β-Glucan-Synthesizing Species

Another interesting approach, together with the extraction of glucans directly from wastes, is the use of the by-products as substrates that can be utilized by species that possess the required metabolic components to produce β-glucans. Linked to this idea, many works have tested vegetal wastes to grow algae or fungal (mushrooms and yeasts) species [41,85,91,93]. 

The olive oil industry generates massive annual volumes of by-products, highlighting olive mill wastewater (OMWW), which is the most released effluent from the production process. This discarded fraction has a great environmental impact due to its high organic and phenolic content, being toxic against plants and soil or marine organisms. Since more than 30 million m^3^ of OMWW per year is produced, novel alternatives must be designed in terms of minimizing the damage and adding value to this waste [85,86]. Thus, OMWW was tested in a wastewater medium to grow *Pleurotus* spp. and *Ganoderma lucidum* that could produce β-glucans [85]. In addition, OMWW also served as growth medium for several mushroom strains (*Pleurotus* spp., *Lentinula edodes*, *Auricularia auricula-judae*, among others) inducing their (1→3)-β-glucan synthase activity [86]. Besides OMWW, olive mill stone waste (OMSW) is another relevant by-product that is formed during olive oil extraction. It is a resultant heterogeneous biomass mainly composed of non-extracted oil and high levels of moisture [87]. Recently, Elipoulos et al. (2022) utilized this material in a solid state fermentation process to cultivate *Pleurotus ostreatus* mushrooms to produce (1→3),(1→6)-β-glucans. In addition, other wastes were utilized in this study with the same purpose, such as oat bran and *Lathyrus clymenum* pericarps [87].

Other mushrooms such as *Cordyceps sinensis* were used to ferment agri-products that are normally discarded such as stale rice, known by its low organoleptic, nutritional and functional properties due to its long-term storage [88]. In this case, the fermented rice showed an increased level of β-glucans due to *C. sinensis* activity and, when different solutions were prepared from the fermented product and administered to healthy Swiss mice, a potent antioxidant capacity was recorded in vivo [88]. Moreover, *Calocybe indica*, commonly known as milky white mushroom, was cultivated using two agro-industrial wastes through submerged fermentation: banana (*Musa x paradisiaca*) peel and de-oiled peanut (*Arachis hypogaea*) cake (a by-product from peanut oil production). The total glucan and β-glucan content of these materials was significantly increased after fermentation [89]. 

Besides mushrooms, other fungal species, particularly yeasts, were cultured using by-products. For instance, *S. cerevisiae* utilized vegetable and fruit wastes, specifically from banana, papaya (*Carica papaya*) and napa cabbage (*Brassica rapa pekinensis*), and β-glucans from the cell walls were extracted by alkali–acid methods leading to fractions with high antioxidant activities in vitro [41]. This yeast was also capable of growing using malva nut (*Scaphium affine*) juice wastewater, producing β-glucans efficiently [90]. Besides *S. cerevisiae*, *Candida utilis* was tested in a study that revalorized potato (*Solanum tuberosum*) juice wastewater. The yeast cultures using this by-product as growth medium led to high yields of (1→3),(1→6)-β-glucans [91]. 

Surprisingly, not many works have been published correlating by-product revalorization and β-glucan production by algal species. In this sense, a study was carried out to implement a zero-waste strategy in bread production. Bread waste was enzymatically hydrolyzed to obtain glucose as a carbon substrate to cultivate *Euglena gracilis* and produce the target compound: paramylon, a (1→3)-β-glucan [93].

## 5. Conclusions, Current Limitations and Future Perspectives

This work reviewed the scientific literature published in relation to the potential revalorization of food and agro-industrial wastes to extract or produce β-glucans with nutraceutical interest. Considering the up-to-date knowledge, it can be concluded that the results obtained linked to the described strategies (using the by-products as both β-glucan sources and as substrates to grow β-glucan-producing organisms) are promising enough to encourage further investigation.

In fact, there are some points that require deeper research since they may be crucial for these purposes and the studies addressing them are still scarce. For instance, it seems important to minimize marine and fluvial wastes, that generate a great impact on the environment, leading to complications such as eutrophication, water, soil and air pollution, toxicity for terrestrial and aquatic organisms, etc. [97]. In this sense, algal species generating these materials can be revalorized since many of them are efficient β-glucan producers and these polysaccharides can be extracted from algal biomass or synthesized in controlled cultures of algal species [25,26]. Moreover, algal wastes can serve as substrates to cultivate other β-glucan producers such as mushrooms or yeasts, since they can utilize algal components for their nutrition [98]. 

Another under-investigated aspect is the characterization of the obtained β-glucans. Most of the studies did not clarify the structure, linkages, molecular weight, solubility, etc. of the extracted or isolated polysaccharides and this lack of information hindered the subsequent tests about the biological activity of the extracted molecules, since structure and function are strongly correlated. Moreover, sometimes the origin of the glucan is not clear, since it is not specified whether it is isolated from the substrate (e.g., a plant) or the organism that bioprocesses the substate (e.g., yeasts or mushrooms).

Finally, the main weakness that must be indicated regarding the current state of the art is the lack of studies addressing the biological activity of the obtained glucans. Just a few studies, most of them targeting antioxidant abilities in vitro and in vivo, can be found, together with some antiproliferative and cytoprotective activities and technological properties [10,27,77,81]. However, it should be noted that hypocholesterolemic and hypoglycemic activities, so widely described and certified for β-glucans, have not been tested yet for those glucans obtained utilizing by-products.

These research lines, together with the design of further in vivo studies including clinical trials, must be carried out to validate the promising results and move the current status to the goal of designing nutraceuticals and functional food based on by-products’ β-glucans.

## Figures and Tables

**Table 1:** Food/natural sources of β-glucans with specific structures.

Natural Source	Examples of Species	β-Glucan Structure/Type	References
Cereals	Oat (*Avena sativa*)	Branched (1→3), (1→4)	[13]
	Wheat (*Titricum aestivum*)		[14]
	Barley (*Hordeum vulgare*)		[15]
	Corn (*Zea mays*)		[16]
	Sorghum (*Sorgum bicolor*)		[17]
Mushrooms	White button (*Agaricus bisporus*)	Linear (1→6); Branched (1→3), (1→6)	[18,19]
	Shiitake (*Lentinula edodes*)		[3]
	Oyster (*Pleurotus ostreatus*)	Linear (1→3); Branched (1→3), (1→6)	[20]
	Porcini (*Boletus edulis*)		[21]
Truffles	Black (*Tuber melanosporum*)	Branched (1→3), (1→6)	[22]
	Summer (*Tuber aestivum*)		[23,24]
	Desert (*Terfezia claveryi*)		[23]
Algae/microalgae	*Euglena gracilis*	Linear (1→3)	[25]
	*Chlorella* spp.		[26]
Yeasts	*Saccharomyces cerevisiae*	Linear (1→6); Linear (1→3); Branched (1→3), (1→6)	[27]
	*Saccharomyces pastorianus*	Branched (1→3), (1→6)	[28]
	*Kluyveromyces marxianus*		[29]
	*Metschnikowia pulcherrima*		[30]
Bacteria	*Levilactobacillus brevis*	Linear (1→3)	[31]
	*Oenococcus oeni*	Branched (1→3), (1→2)	[32]
	*Lactobacillus diolivorans*		
	*Pediococcus parvulus*		

**Table 2:** Extraction methods to obtain β-glucans from food/natural sources.

Extraction Method	Temperature Range (°C)	Usually Utilized Solvents	References
Conventional solid–liquid	25–100	Water, ethanol	[43,44,45,46]
Alkaline solid–liquid	22–80	Alkaline solutions	[3,47,48]
Enzyme-assisted	50–96	Water, buffer solutions	[49,50,51]
Microwave-assisted	50–180 (750–850 W)	Water, ethanol	[52,53,54,55]
Ultrasound-assisted	20–90	Water, ethanol	[56,57,58]
Pressurized liquid	50–200	Water, ethanol	[22,53,56,59,60,61]
Pulsed electric field-assisted	4–85	Water	[62,63]

**Table 3:** Examples of food by-products and agro-industrial wastes as a source of β-glucans with potential biological or technological functionalities.

Raw Material	Obtained β-Glucan/s	Functionality	Reference/s
Sedimented wine yeast	1→3, 1→6	Water- and fat-binding	[75,76]
Spent beer yeast	1→3, 1→6	Water-holding, fat replacer, antioxidant, cytoprotective	[10,27,77]
Barley by-products	-	-	[61,78]
Oat mill waste	-	-	[79]
Walnut husks	-	-	[80]
Pomegranate peel	-	Antioxidant, antiproliferative	[81]
Bean pods	-	-	[82]
Mushroom (*Agaricus bisporus*) by-products (caps and stalks)	-	-	[57]
Mushroom (*Pleurotus pulmonarius*) stalks	-	Antioxidant	[83,84]
Olive mill wastewater/mushrooms strains	-	Antioxidant	[85,86]
Olive mil stone waste/*Pleurotus ostreatus*	1→3, 1→6	-	[87]
Oat bran/*Pleurotus ostreatus*	1→3, 1→6	-	[87]
*Lathyrus clymenum* pericarps/*Pleurotus ostreatus*	1→3, 1→6	-	[87]
Stale rice/*Cordyceps sinensis*	-	Antioxidant	[88]
Banana peel/*Calocybe indica*)	-	-	[89]
De-oiled groundnut cake/*Calocybe indica*	-	-	[89]
Napa cabbage, banana and papaya wastes/*Saccharomyces cerevisiae*	-	Antioxidant	[41]
Malva nut juice wastewater/*Saccharomyces cerevisiae*	-	-	[90]
Potato juice water/*Candida utilis*	1→3, 1→6	-	[91,92]
Bread waste/*Euglena gracilis*	Paramylon, 1→3	-	[93]

## Data Availability

Data sharing not applicable.

## Abbreviations

European Food Safety Authority (EFSA), lactic acid bacteria (LAB), 3-hydroxy-3-methylglutaryl-coenzyme A reductase (HMGCR), olive mill wastewater (OMWW), olive mill stone waste (OMSW).
